# Tunable Terahertz Optical Bistability Enabled by Synergistic Topological Edge States and Weyl Semimetal

**DOI:** 10.3390/nano16181144

**Published:** 2026-09-12

**Authors:** Zhiheng Li, Zean Shen, Liuxin Qian, Zhiwei Zheng, Leyong Jiang

**Affiliations:** 1 School of Physics and Electronics, Hunan Normal University, Changsha 410081, China; 2 Key Laboratory of Physics and Devices in Post-Moore Era, College of Hunan Province, Changsha 410081, China

**Keywords:** optical bistability, topological edge states, weyl semimetal, photonic crystal heterostructure

## Abstract

Low-threshold optical bistability (OB) holds significant application prospects in various fields. In this work, we theoretically propose a one-dimensional photonic crystal heterostructure incorporating a Weyl semimetal (WSM) layer. The design synergistically combines the local field enhancement associated with topological edge states (TESs) and the strong intrinsic nonlinearity of WSM. Through parameter optimization, a tunable OB phenomenon with a threshold of 10^5^ V/m for both the incident electric field and the transmitted electric field is achieved in the terahertz regime. Furthermore, key characteristics of the OB, including the switching threshold and hysteresis loop width, can be flexibly manipulated by adjusting parameters such as the angle of incidence, the incident wavelength, and the thickness of the WSM layer. We believe that the proposed scheme may provide useful theoretical guidance for future studies of low-threshold nonlinear phenomena and terahertz photonic functionalities based on WSM.

## 1. Introduction

Optical bistability (OB) is a phenomenon in which a single input light intensity corresponds to two distinct output intensities. When an OB phenomenon occurs, the relationship between the input and output light intensities exhibits a hysteresis loop. These two output light intensities represent two stable states, commonly referred to as the high and low output states. Owing to its bistable characteristics, OB exhibits extensive applications in optical memory [1], biosensing [2], optical communications [3], all-optical switching [4] and optical differential operation [5]. Although the physical mechanisms and basic theories of OB are well established, practical applications of OB-based devices still face several limitations. In particular, the relatively high threshold required for OB generation makes it difficult to satisfy the demands of realistic application scenarios. Consequently, minimizing the OB threshold has become a critical objective in the study of OB. Various strategies have been proposed to lower the OB threshold; a representative example is the Fabry–Perot cavity, which enhances the intracavity electric field through resonance, thereby lowering the OB threshold [6]. In addition to structural design, mechanisms such as surface plasmon polaritons [7], bound states in the continuum [8], topological edge states (TESs) [9], Fano resonance [10,11], and optical Tamm states [12] can also induce strong local field enhancement, leading to an effective reduction in the OB threshold. Furthermore, the identification of materials with large, and preferably extremely large, nonlinear optical properties is crucial for reducing the OB threshold. In recent years, numerous studies have focused on OB based on nonlinear materials such as graphene [13,14] and Dirac semimetals [15], successfully achieving low-threshold and controllable OB. However, these schemes also have limitations. Taking graphene as an example, its two-dimensional properties have caused a bottleneck in large-area and high-quality preparation, which slows down the research progress of optical bistable devices based on graphene.

Recently, the Weyl semimetal (WSM) has attracted considerable attention as a material with unique topological properties. Its distinctive characteristics have drawn widespread interest across multiple fields, including optics, materials science, and chemistry. In the field of optics, certain properties of WSM are particularly notable. The WSM exhibits pronounced nonlinear optical responses, such as third-harmonic generation [16]. Almutairi et al. investigated the nonlinear response of WSM using dynamic equations, calculating the intensities of nonlinear four-wave mixing signals in both transmitted and reflected geometries. Their results showed that doped bulk WSM exhibits extremely high third-order nonlinearity, accompanied by substantial absorption loss [17]. Lai et al. reported the third-order nonlinear Hall effect in Td-MoTe_2_, a WSM material, and demonstrated that this effect is directly related to the Berry connection polarizability tensor [18]. Tilmann et al. investigated the linear and third-order nonlinear optical properties of N_b_P films using bulk single crystals as references, and observed strong third-harmonic generation from the films [19]. Moreover, the presence of a finite magnetic field can significantly enhance the nonlinear optical response of WSM [20]. In the field of OB, some researchers have recognized the outstanding properties of WSM and conducted studies based on them. For example, in 2023, Yin et al. incorporated a WSM defect layer into an asymmetric one-dimensional photonic crystal, combining the strong third-order nonlinear response of the WSM with the local field enhancement of the defect mode to achieve low-threshold OB absorption [21]. In 2024, Yin et al. further investigated non-reciprocal OB in magnetic WSM sandwich structures [22].

In this work, we theoretically propose a one-dimensional photonic crystal (1D PhC) heterostructure incorporating a WSM to achieve low-threshold, tunable OB in the terahertz band. By embedding the WSM at the interface of two 1D PhCs, the strong nonlinear optical response of the WSM is combined with the TESs supported by the heterogeneous photonic crystal structure. This configuration significantly enhances the local electric field at the WSM photonic crystal interface, enabling OB with a transmitted electric field threshold on the order of 10^5^ V/m. In addition, both the OB threshold and the width of the hysteresis loop can be dynamically tuned by varying parameters such as the incident angle, incident wavelength, and WSM layer thickness. In particular, the threshold width of OB can be flexibly adjusted simply by changing the incident wavelength, allowing controlled switching between large and small threshold widths. The proposed structure provides a conceptual, theoretical reference scheme for investigating controllable OB arising from the interplay between TESs enhancement and the nonlinear response of WSM.

## 2. Theoretical Model and Method

**Figure 1:**
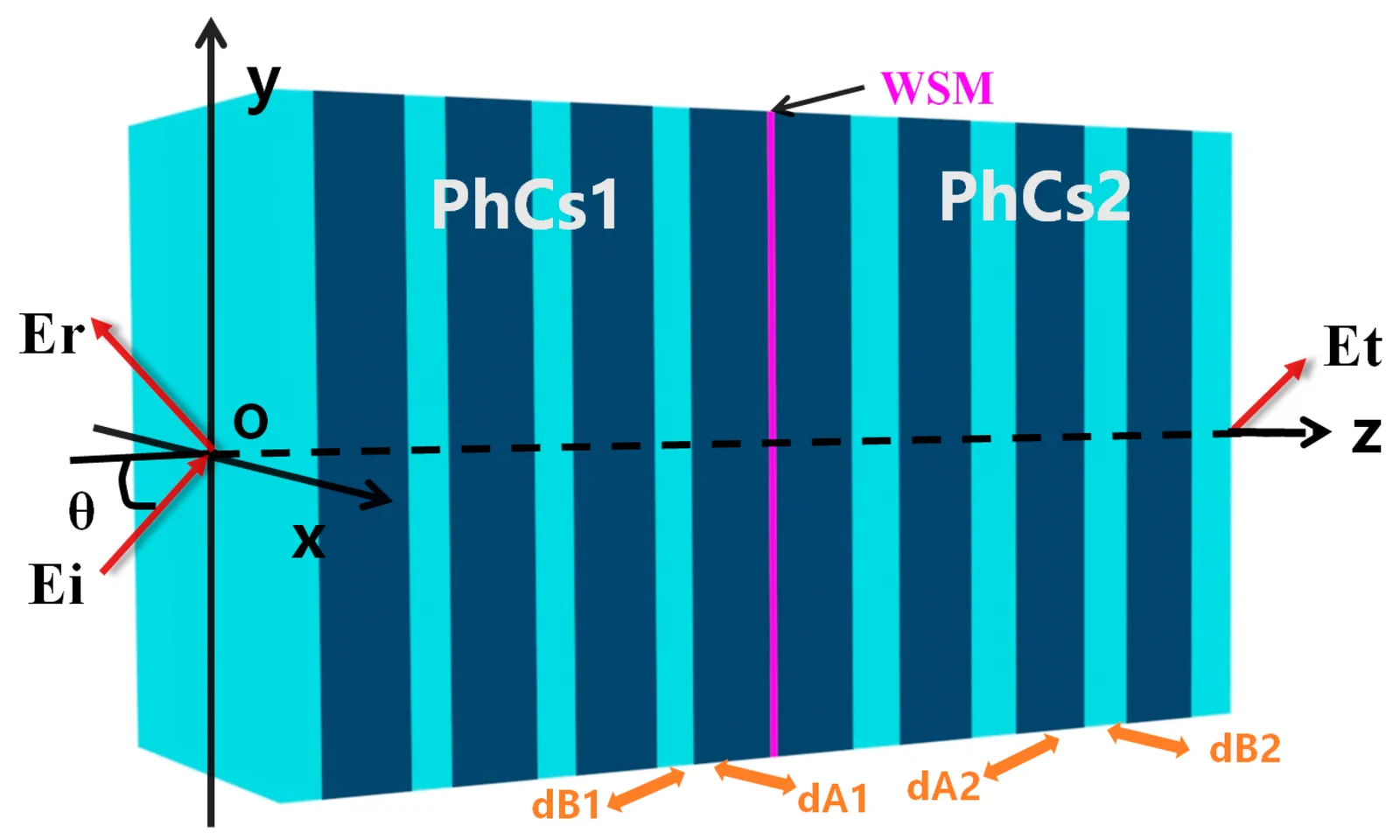
Schematic diagram of the WSM/PhCs structure, where the WSM layer is embedded between PhCs1 and PhCs2. A plane wave with amplitude $E_{i}$ is incident on the structure at an angle θ.

The structure considered in this work consists of two 1D PhCs, denoted as PhCs1 and PhCs2, with a WSM embedded at their interface, as shown in Figure 1. Our core objective in considering the combination of two types of PhCs is to excite TESs throughout the entire structure, thereby creating conditions for significant local field enhancement. The excitation of TESs has the potential to produce significant local field enhancement. At this point, embedding WSM between two 1D PhCs can significantly increase the nonlinear coefficient of WSM and create conditions for low-threshold OB. Here, both PhCs1 and PhCs2 are composed of alternating layers of materials A (TPX) and B (TiO_2_). For clarity, the A and B layers in PhCs1 are denoted as A1 and B1, respectively, while those in PhCs2 are denoted as A2 and B2. The refractive indices of medium A are set to n_A1_ = n_A2_ = 1.46, while those of medium B are set to n_B1_ = n_B2_ = 2.82. In PhCs1, the thicknesses of layers A and B are chosen as d_A1_ = 270 μm and d_B1_ = 121 μm, respectively. In PhCs2, the corresponding thicknesses are d_A2_ = 275 μm and dB2 = 170 μm, while the thickness of the WSM layer is set to 200 nm. Generally speaking, the selection of the thickness of each dielectric layer is related to the wavelength of the incident wave and the refractive index of the dielectric. That is, *d_A_* = λ/4*n_A_* and *d_B_* = λ/4*n_B_*. However, in this manuscript, we did not fully adhere to this relationship. This is mainly to control the position of the band gap between two photonic crystals in order to create positive factors for TESs. At present, micro-nanofabrication and measurement technology based on multilayer dielectric structure is relatively mature. Theoretically, it is feasible to use existing nanomanufacturing technology.

In the strong degeneracy/low-temperature limit, the linear conductivity of WSM can be expressed as [17]

(1) $$\sigma_{\left(\omega \right)}^{\left(1\right)}=\frac{e^{2}v_{F}P_{F}^{2}g_{s}g_{\omega }}{6\pi^{2}\hbar^{3}(\gamma -i\omega )}.$$

The specific parameter values employed in this work are adopted from previously reported theoretical studies of WSM and are selected to lie within the parameter ranges commonly used in the literature for investigating their terahertz optical responses. In Equation (1), e denotes the electron charge and ω represents the angular frequency of the incident light. The parameter $V_{F}$ = 10^6^ m/s is the Fermi velocity, while $E_{F}$ = 4 meV corresponds to the Fermi energy of the WSM. The quantity $P_{F}=E_{F}/V_{F}$ denotes the magnitude of the electron momentum. The parameter $g_{\omega }$ is the degeneracy factor associated with the Weyl nodes; in this work, $g_{\omega }$ is set to 4, corresponding to two pairs of Weyl points. Larger values of $g_{\omega }$ are expected to result in stronger nonlinearity [17]. The parameter $g_{s}$ is a degeneracy factor describing the spin degree of freedom. In magnetic WSM with broken time-reversal symmetry, spin polarization leads to $g_{s}$ = 1, whereas in non-magnetic WSM with preserved time-reversal symmetry, $g_{s}=2$. In this work, $g_{s}$ = 2 is adopted. Finally, ℏγ = 5 meV and ℏ is the reduced Planck constant.

The WSM considered in this paper is an isotropic material, and its linear dielectric permittivity is given by [17]:

(2) $$\varepsilon_{1}=\varepsilon_{b}+\frac{i4\pi \sigma^{\left(1\right)}}{\varepsilon_{0}\omega }.$$

Here, $\varepsilon_{b}$ = 10 denotes the background dielectric constant, and $\varepsilon_{0}$ represents the vacuum permittivity. It should be noted that “isotropy” is only a local approximation of the low-energy effective theory. The “isotropy” of WSM is a local property under specific theoretical approximations, not a macroscopic intrinsic property of the material.

The third-order conductivity of the WSM can be expressed as [17]

(3) $$\sigma_{\left(\omega \right)}^{\left(3\right)}=\frac{e^{4}v_{F}g_{s}g_{\omega }}{15\pi^{2}\hbar^{3}(-i\omega +\gamma )}\left[\frac{\frac{1}{i\omega +\gamma }+\frac{1}{-i\omega +\gamma }}{\gamma }+\frac{1}{\left(-i2\omega +\gamma )(-i\omega +\gamma \right)}\right].$$

In theory, a complete nonlinear response should correspond to all nonlinear coefficients of third order or higher. However, generally speaking, considering that the high-order nonlinear coefficients of nonlinear materials are very small, the impact on the entire nonlinear response is negligible. Therefore, in general, we only consider the influence of third-order nonlinear conductivity. Based on this consideration, the refractive index of the WSM includes both linear and nonlinear components and can be written as

(4) $$n_{C}=\sqrt{\varepsilon_{1}+\chi^{\left(3\right)}|E_{iside}{|}^{2}}$$

Here, $E_{iside}$ represents the electric field intensity at the incident interface of the WSM layer, composed of the incident electric field $E_{i}$ and the reflected electric field $E_{r}$. Its value varies with the position within the WSM layer. The calculation of $E_{iside}$ will be addressed later in the discussion on the treatment of WSM as a nonlinear material.

$\chi^{\left(3\right)}$ denotes the third-order susceptibility of the WSM, and its expression is given by [17]:

(5) $$\chi^{\left(3\right)}=\frac{i\sigma^{\left(3\right)}}{\varepsilon_{0}\omega }$$

It is necessary to note that the description of OB is mainly from the perspective of the electric field, that is, the relationship between the transmitted or reflected electric field and the incident electric field. In addition, threshold width is also an important indicator. Of course, in some works, it is also customary to describe the relationship between transmitted or reflected light intensity and incident light intensity. The conversion relationship between the two can be expressed as $I=c\varepsilon_{0}n{\left|E_{in}\right|}^{2}/2$. Among them, c represents the speed of light, $\varepsilon_{0}$ represents the dielectric constant in vacuum, and n represents the refractive index. The relationship among the incident, reflected, and transmitted electric fields can be determined using the transfer matrix method. In this work, the transmittance and reflectance of the composite structure incorporating the WSM under TE polarization are calculated via the transfer matrix method. The material layers, including the WSM layer, are assumed to be parallel to the XY plane, while the electromagnetic field propagates along the *Z*-axis. Under these conditions, the transfer matrix for each material layer can be expressed as follows:

(6) $$M_{j}=\left(\begin{array}{cc} \cos(k_{jz}d_{j}) & -\frac{i}{\eta_{j}}\sin(k_{jz}d_{j})\\ -i\eta_{j}\sin(k_{jz}d_{j}) & \cos(k_{jz}d_{j}) \end{array}\right).$$

In Equation (6), $k_{jz}$ is the wave vector component along the *Z*-axis for the j-th layer, given by $k_{jz}=n_{j}\frac{\omega }{c}\cos(\theta_{j})$, where $n_{j}$ is the refractive index of the layer, $\theta_{j}$ is the refraction angle, and $d_{j}$ is the layer thickness. $\eta_{j}=\sqrt{\varepsilon_{0}/\mu_{0}}n_{j}\cos(\theta_{j})$ represents the optical admittance under TE polarization. In this expression, $\varepsilon_{0}$ denotes the vacuum permittivity, and $\mu_{0}$ is the vacuum permeability.

However, for the nonlinear WSM, as indicated by Equation (4), its refractive index is strongly dependent on the nearby electric field. Consequently, the standard transfer matrix is no longer applicable. In previous studies of OB involving nonlinear materials such as graphene and Dirac semimetal, the nonlinear response was treated by solving Maxwell’s equations together with the appropriate boundary conditions. In this approach, the nonlinearity is incorporated by modifying the boundary conditions to obtain the transmission and reflection coefficients of the nonlinear layer, which are then combined with the transfer characteristics of the linear layers to determine the overall transmittance and reflectance of the structure. Currently, there are several calculation algorithms for OB, corresponding to different structures, scenarios, and limitations. For relatively simple few-layer structures with nonlinear materials as substrates, the bistable curve, transmittance, and reflectance of the entire structure can only be achieved by solving the nonlinear Maxwell’s equations. For multilayer structures containing nonlinear materials, where the bistable curve, transmittance and reflectance of the entire structure can be achieved through a nonlinear transmission matrix. For multilayer structures containing two-dimensional materials, the bistable curve, transmittance and reflectance of the entire structure can be achieved through the iteration of Maxwell’s equations and boundary conditions. For non-layered structures such as metasurfaces, gratings, and other complex structures, the transmittance and reflectance of the entire structure need to be achieved through numerical simulation software. In this study, the treatment of the nonlinear WSM differs fundamentally from previous approaches. Instead of introducing nonlinearity through modified boundary conditions, we adopt a layer-wise approximation method. Specifically, the WSM layer is divided into a sufficiently large number of thin sublayers along the propagation direction. Within each sublayer, the electric field intensity is assumed to be approximately constant, and the field at the right boundary of the sublayer is taken as the representative value [21].

Specifically, we first calculate the transfer matrix $M_{PhCs2}$ of the right photonic crystal, $M_{PhCs2}={\left(M_{A2}M_{B2}\right)}^{4}$. The electric field at the interface between the WSM layer and PhCs2 satisfies the following relationship:

(7) $$E_{iside}=M_{PhCs2}(1,1)*E_{t}+M_{PhCs2}(1,2)*E_{t}*\eta_{N}$$

In Equation (7), $E_{iside}$ denotes the electric field at the incident-side interface of the WSM layer, $E_{t}$ is the electric field at the transmission side of PhCs2, and $\eta_{N}$ represents the optical admittance of the transmission-side medium. In this work, for the composite structure PhCs1 + WSM + PhCs2, both the incident and transmission media are assumed to be vacuum.

If the WSM layer is divided into n sublayers along the propagation direction, the transfer matrix corresponding to the N-th sublayer of the WSM is given by:

(8) $$M_{n}=\left(\begin{array}{cc} \cos(k_{nz}d_{n}) & -\frac{i}{\eta_{n}}\sin(k_{nz}d_{n})\\ -i\eta_{n}\sin(k_{nz}d_{n}) & \cos(k_{nz}d_{n}) \end{array}\right).$$

In Equation (8), $k_{nz}$ is the wave vector of the N-th WSM sublayer along the direction perpendicular to the incident wave, $d_{n}$ is the thickness of this sublayer, and $\eta_{n}$ represents the optical admittance of the WSM sublayer under TE polarization.

Thus, the transfer matrix corresponding to the entire WSM layer can be expressed as

(9) $$\left[\begin{array}{c} E_{1}\\ H_{1} \end{array}\right]=M_{1}M_{2}M_{3}\ldots M_{n-2}M_{n-1}M_{n}M_{PhCs2}\left[\begin{array}{c} E_{n+1}\\ H_{n+1} \end{array}\right],$$

Here, $E_{1}$ and $H_{1}$ denote the electric and magnetic fields at the incident interface of the WSM layer, respectively, while $E_{n+1}$ and $H_{n+1}$ represent the electric and magnetic fields at the exit interface of the PhCs2. At the incident side, both the incident and reflected electric fields are present, whereas no reflected field exists at the transmission side.

The transfer matrix of the entire WSM layer can be expressed as

(10) $$M_{WSM}=M_{1}M_{2}M_{3}\ldots M_{n-2}M_{n-1}M_{n}$$

To validate the nonlinear treatment of the WSM layer, we note that the layer-wise approximation method employed in this work has been widely adopted in studies of nonlinear photonic structures. In this approach, the nonlinear medium is discretized into sufficiently thin sublayers such that the electric field within each sublayer can be considered approximately uniform. The convergence of this method is ensured when the number of sublayers is sufficiently large.

At this point, the total transfer matrix of the entire composite structure can be written as $M={\left(M_{B1}M_{A1}\right)}^{4}M_{WSM}{\left(M_{A2}M_{B2}\right)}^{4}$.

After obtaining the total transfer matrix, the reflection coefficient of the entire structure can be calculated as follows:

(11) $$r=\frac{M_{11}\eta_{0}+M_{12}\eta_{0}\eta_{N}-M_{21}-M_{22}\eta_{N}}{M_{11}\eta_{0}+M_{12}\eta_{0}\eta_{N}+M_{21}+M_{22}\eta_{N}}.$$

Meanwhile, the transmission coefficient of the entire structure can be obtained as

(12) $$t=\frac{2\eta_{0}}{M_{11}\eta_{0}+M_{12}\eta_{0}\eta_{N}+M_{21}+M_{22}\eta_{N}}.$$

Based on the reflection coefficient r and the transmission coefficient t of the composite structure, the reflectance R and transmittance T can be calculated. Furthermore, using the relationship between the transmitted electric field $E_{t}$ and the incident electric field $E_{i}$, the modulus of the transmitted electric field can be obtained as

(13) $$\left|E_{t}|=|E_{i}|\times |t\right|$$

The modulus of the reflected electric field is

(14) $$\left|E_{r}|=|E_{i}|\times |r\right|$$

We divide the WSM layer into several sublayers, with the fundamental purpose of dealing with the field strength distribution problem of nonlinear materials. By subdividing the nonlinear layer of thickness into *N* sublayers and approximating the electric field strength as a constant within each sublayer, the transmission matrix of each sublayer can adopt the same form as that of a linear medium. This is a numerical processing method, rather than a physical notion that each sublayer is an independent “new material” that satisfies the bulk approximation. Each sublayer is still a part of the large WSM material; Layering is only for discretizing the field strength changes in space, facilitating iterative solving of nonlinear transmission problems. Physically speaking, as long as the total thickness satisfies the bulk approximation condition, layering does not change this, because each sublayer is still a local part of the same bulk material, and its microscopic electronic structure has not changed due to numerical segmentation. Therefore, we believe that there is no need to discuss the effectiveness of the volume approximation layer by layer separately. It is only necessary to ensure that the total thickness meets the conditions and that the thickness of the sublayers is not small enough to enter quantum confinement or areas with significant size effects (such as not less than a few nanometers). In our calculations, this condition can be satisfied.

## 3. Results and Discussion

**Figure 2:**
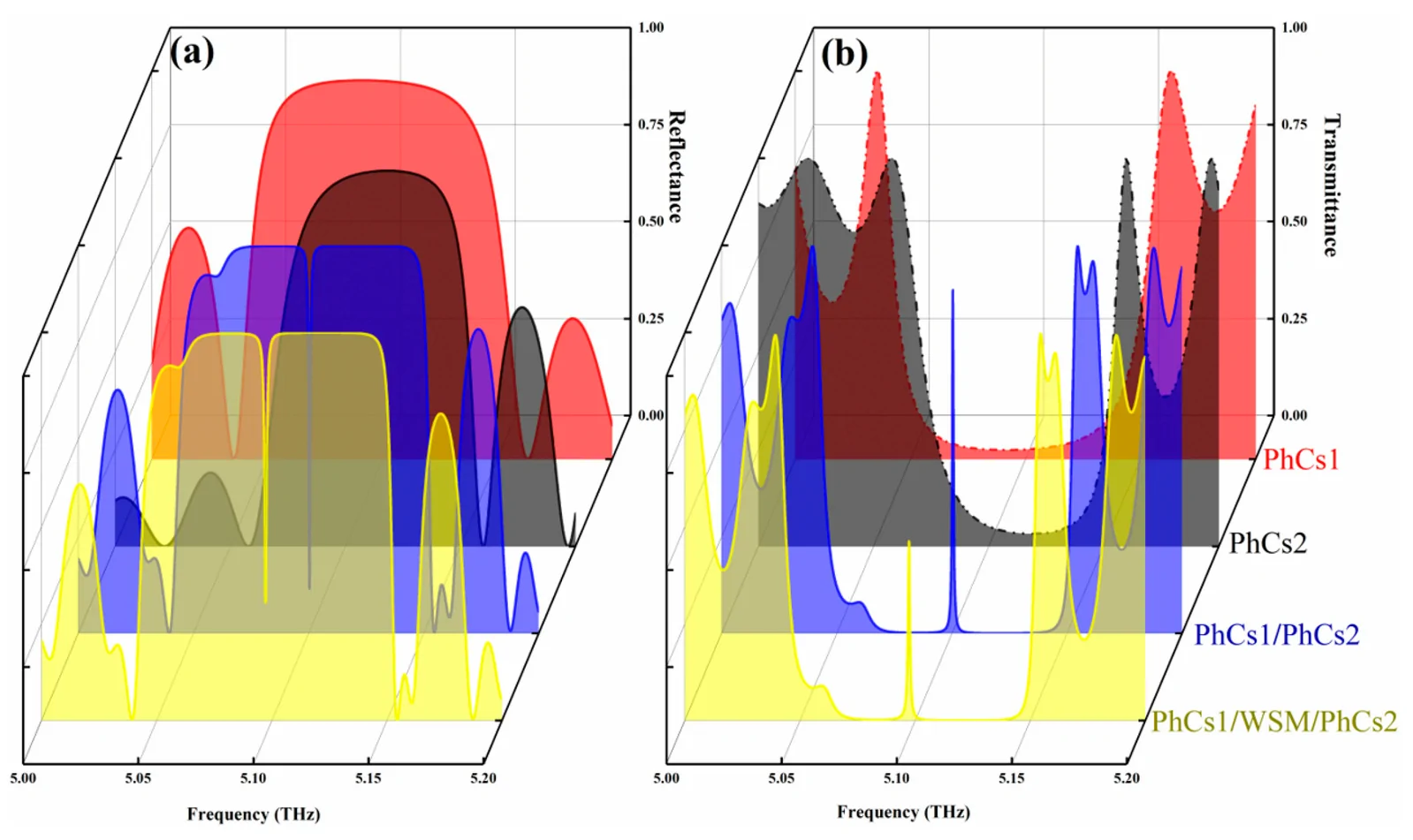
(**a**) Reflection spectra of PhCs1 (red curve), PhCs2 (black curve), PhCs1/PhCs2 (blue curve), and PhCs1/WSM/PhCs2 (yellow curve). (**b**) Transmission spectra of PhCs1 (red curve), PhCs2 (black curve), PhCs1/PhCs2 (blue curve), and PhCs1/WSM/PhCs2 (yellow curve).

In this section, we first characterize the bandgap properties of a single PhC and the composite structure based on the transfer matrix method. In addition to the transmittance and reflectance, the absorption of the structure is also evaluated to ensure physical consistency. The absorption is defined as A=1−R−T. Our calculations confirm that energy conservation is satisfied in all simulations and the absorption mainly originates from the imaginary part of the WSM permittivity. The presence of absorption plays a crucial role in determining the balance between nonlinear enhancement and loss, which directly affects the emergence of optical bistability. The corresponding reflection and transmission spectra are presented in Figure 2a,b, respectively. It can be seen that both PhCs1 (red curve) and PhCs2 (black curve) exhibit wide bandgaps centered around 5.1 THz (5.06–5.16 THz), with the bandgap of PhCs1 being slightly wider than that of PhCs2. The presence of a common bandgap satisfies the necessary condition for the excitation of TESs. In addition, Figure 2a,b also present the spectra of the combined structure PhCs1/PhCs2 (blue curve) and the composite structure PhCs1/WSM/PhCs2 (yellow curve) after the introduction of the WSM layer. Since the frequency of the TESs in the photonic crystal depends on the incident angle, the incident angle is fixed at 0° in this work. The results indicate that the PhCs1/PhCs2 structure exhibits sharp transmission peaks and corresponding reflection minima around 5.1 THz. Notably, after the introduction of the WSM layer, the amplitudes of both the transmission peaks and the reflection minima of the composite structure are reduced, accompanied by slight spectral shifts. It can be seen that the addition of WSM would not affect the excitation of TESs. However, its nonlinearity and tunability can provide a basis for the realization and manipulation of OB. It is worth noting that although the defect modes of PhCs can also produce a similar transmission peak, they are different in essence. When there are defects in the PhCs, a new defect-mode conduction band will appear in the bandgaps. The propagation of frequency occurs in the frequency range where transmission was completely prohibited before, and it shows a sharp transmission peak in the macro view.

**Figure 3:**
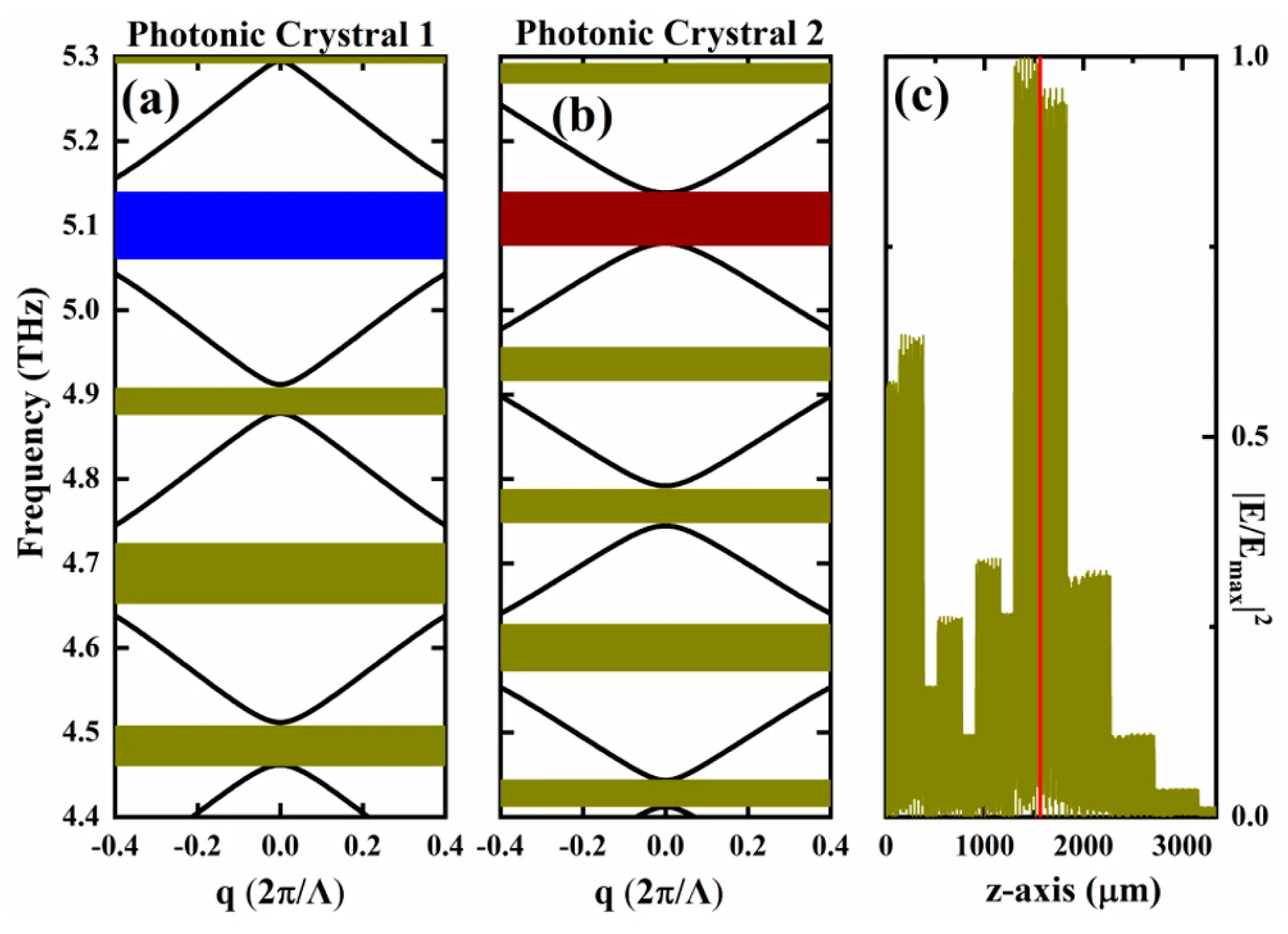
The band structures of (**a**) PhCs1 and (**b**) PhCs2 and (**c**) electric field distribution of the composite structure.

Subsequently, the electric field distribution in the vicinity of the WSM layer is analyzed. As shown in Figure 3c, pronounced electric-field localization is observed at the interface between PhCs1 and PhCs2; the region marked by the red line in Figure 3c corresponds to the position of the WSM layer. It is well established that when two PhCs share a common bandgap within the same frequency range and the cumulative Zak phases of the two PhCs within this bandgap differ by an odd multiple of π, TESs can be excited at the interface of the photonic crystal heterostructure [23,24]. Following the approach in Ref. [24], the dispersion relation of a one-dimensional photonic crystal can be expressed as

(15) $$\cos(q\wedge )=\cos k_{a}d_{a}\cos k_{b}d_{b}-\frac{1}{2}(\frac{z_{a}}{z_{b}}+\frac{z_{b}}{z_{a}})\sin k_{a}d_{a}\sin k_{b}d_{b},$$

In this equation, q denotes the Bloch wave vector; $\wedge =d_{a}+d_{b}$ represents the lattice constant (the total thickness of one unit cell); $k_{a}$ and $k_{b}$ are the wave vectors in materials A and B, respectively; $d_{a}$ and $d_{b}$ denote the thicknesses of materials A and B, respectively; and $z_{a}$ and $z_{b}$ are the characteristic impedances of materials A and B. If TESs exist at the interface between PhCs1 and PhCs2, the condition $z_{s,1}+z_{s,2}=0$ must be satisfied, indicating that the topological properties of the two PhCs within the shared bandgap are opposite. The surface impedance $z_{s}$ is determined by the geometric phase of the photonic band, namely the Zak phase, and can be expressed as [24]

(16) $$\theta_{n}^{\mathrm{Zak}}=\int_{-\pi /\Lambda }^{\pi /\Lambda }\left[i\int_{\mathrm{unit}\;\mathrm{cell}}dz\varepsilon (z)u_{n,q}^{*}(z)\partial_{q}u_{n,q}(z)\right]dq$$

In Equation (16), $i\int_{\mathrm{unit}\;\mathrm{cell}}dz\varepsilon (z)u_{n,q}^{*}(z)\partial_{q}u_{n,q}(z)$ is the Berry connection, ε(z) denotes the dielectric function, and $u_{n,q}^{*}(z)$ is the periodic-in-cell part of the Bloch electric field eigenfunction of a state on the nth band with wave vector q. As shown in Figure 3a,b, PhCs1 exhibits five bandgaps within the incident frequency range from 4.4 THz to 5.3 THz, whereas PhCs2 presents six bandgaps within the same frequency range. Notably, an overlap exists between the blue-shaded bandgap of PhCs1 spanning 5.06–5.13 THz and the red-shaded bandgap of PhCs2 covering 5.08–5.13 THz. This overlapping region constitutes the common bandgap shared by PhCs1 and PhCs2.

According to the winding-number difference criterion, whether TESs can be excited at the interface between two topologically trivial materials is determined by the difference in their bulk topological invariants. When the winding numbers of the two materials differ, TESs emerge at their interface [25]. In the work of Li et al., the relationship between the winding-number difference and the existence of TESs was experimentally verified [26]. The Zak phase is a topological invariant that characterizes the band topology of a one-dimensional system. After taking the modulus with respect to 2π, its value can be either 0 or π, and the system corresponds to a topologically trivial or nontrivial phase, respectively. By evaluating the Zak phases, the winding numbers of PhCs1 and PhCs2 can be obtained. Consequently, it can be determined that when the incident frequency lies within the common bandgap range of the two PhCs (5.08–5.13 THz), TESs are excited at the interface between PhCs1 and PhCs2. The existence of TESs is confirmed by the presence of a sharp transmission peak within the common bandgap and strong field localization at the interface, which are well-established signatures of TESs in one-dimensional photonic crystal heterostructures.

**Figure 4:**
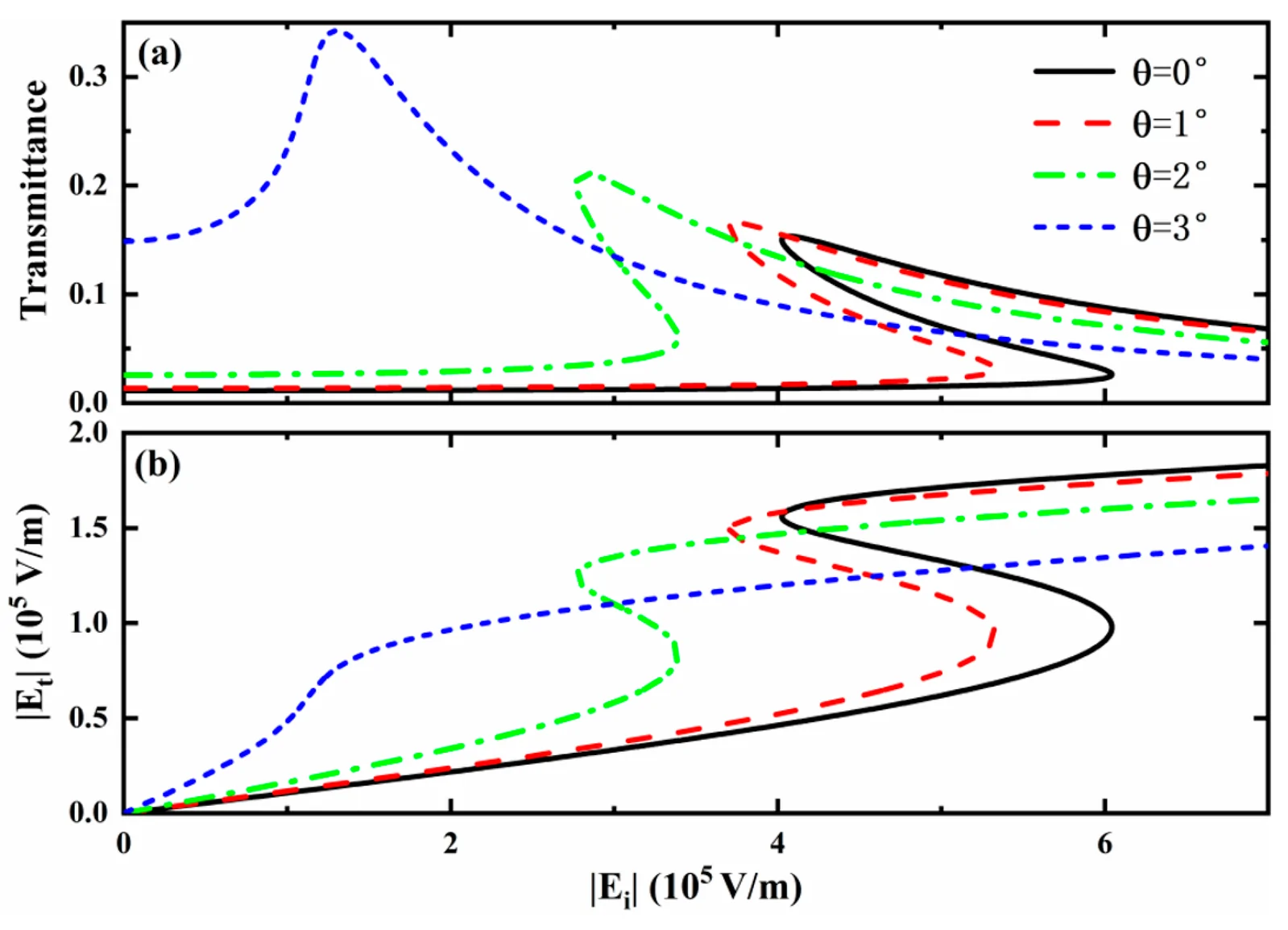
The dependence of (**a**) the transmittance and (**b**) the transmitted electric field on the incident electric field under different incident angles. The incident frequency is fixed at 5.1004 THz, and the WSM layer thickness is set to 200 nm.

In fact, in addition to the TESs observed at the interface between PhCs1 and PhCs2 when the incident frequency is around 5.1 THz, we also find that TESs can be excited at this interface when the incident frequency is around 1 THz. However, when the incident frequency is set to 1 THz, the expected OB does not occur. To clarify this phenomenon, it is necessary to revisit the characteristics of the WSM discussed earlier. The first-order conductivity of the WSM is denoted by Equation (1), the linear dielectric constant by Equation (2), and the third-order nonlinear conductivity by Equation (3). When the incident frequency is 1 THz, the corresponding angular frequency is $\omega \approx 6.2832\times 10^{12}\;\mathrm{rad}/s$, yielding $|\sigma_{\left(\omega \right)}^{\left(1\right)}|\approx 122.7\;S/m$ and $\varepsilon_{1}\approx 10+27.72i$. When the incident frequency is 5.1 THz, the corresponding angular frequency is $\omega \approx 3.204\times 10^{13}\;\mathrm{rad}/s$, with the first-order conductivity and linear dielectric constant given by $|\sigma_{\left(\omega \right)}^{\left(1\right)}|\approx 36.72\;S/m$ and $\varepsilon_{1}\approx 10+1.626i$, respectively. Calculations indicate that the modulus $\left|\sigma_{\left(\omega \right)}^{\left(3\right)}\right|$ of the third-order nonlinear conductivity of the WSM at an incident frequency of 1 THz is larger than that at 5.1 THz, implying a stronger third-order nonlinear response at 1 THz. However, the emergence of OB requires not only sufficiently strong nonlinearity but also sufficiently low linear loss so that the refractive-index modulation induced by the nonlinear effect can play a dominant role. In this scheme, although the $\left|\sigma_{\left(\omega \right)}^{\left(3\right)}\right|$ of the WSM is larger at 1 THz, the imaginary part of the corresponding linear dielectric constant is also significantly increased, leading to enhanced absorption and a strong suppression of the effective nonlinear response. As a result, the nonlinear-induced refractive index modulation cannot overcome the linear loss, and the threshold condition for OB is not satisfied. Consequently, OB does not occur at this frequency. This phenomenon highlights the importance of simultaneously considering nonlinear enhancement and absorption loss when investigating OB in WSM-based photonic structures, since a stronger nonlinear response alone does not necessarily guarantee the emergence of OB. In certain cases, a moderate nonlinear coefficient combined with a low-loss environment may be more favorable for the emergence of nonlinear optical phenomena than a strongly nonlinear material accompanied by significant absorption losses. Then, using the method for calculating OB in composite photonic crystals incorporating WSM described in the previous section, we investigated the influence of the incident angle on OB under fixed incident frequency and WSM thickness. The corresponding results are shown in Figure 4a,b.

Figure 4 reveals the sensitive response of the OB induced by the composite structure after the introduction of the WSM as the incident angle varies. When the incident angle is 0°, a distinct OB hysteresis curve is observed. As the incident angle increases from 0° to 2°, the upper threshold $|E_{i}{|}_{up}$, lower threshold $|E_{i}{|}_{down}$, and threshold width of the OB all decrease, whereas the incident electric field threshold level of the OB remains constant at 10^5^ V/m throughout. When the incident angle increases to 3°, the hysteresis curve abruptly transitions to a single-valued curve. These results indicate that variation in the incident angle/variations in the incident angle can effectively regulate the OB behavior. As the incident angle continues to increase, the phase-matching conditions of the TESs are disrupted, leading to a mismatch of the OB critical point and, consequently, the disappearance of the OB phenomenon.

**Figure 5:**
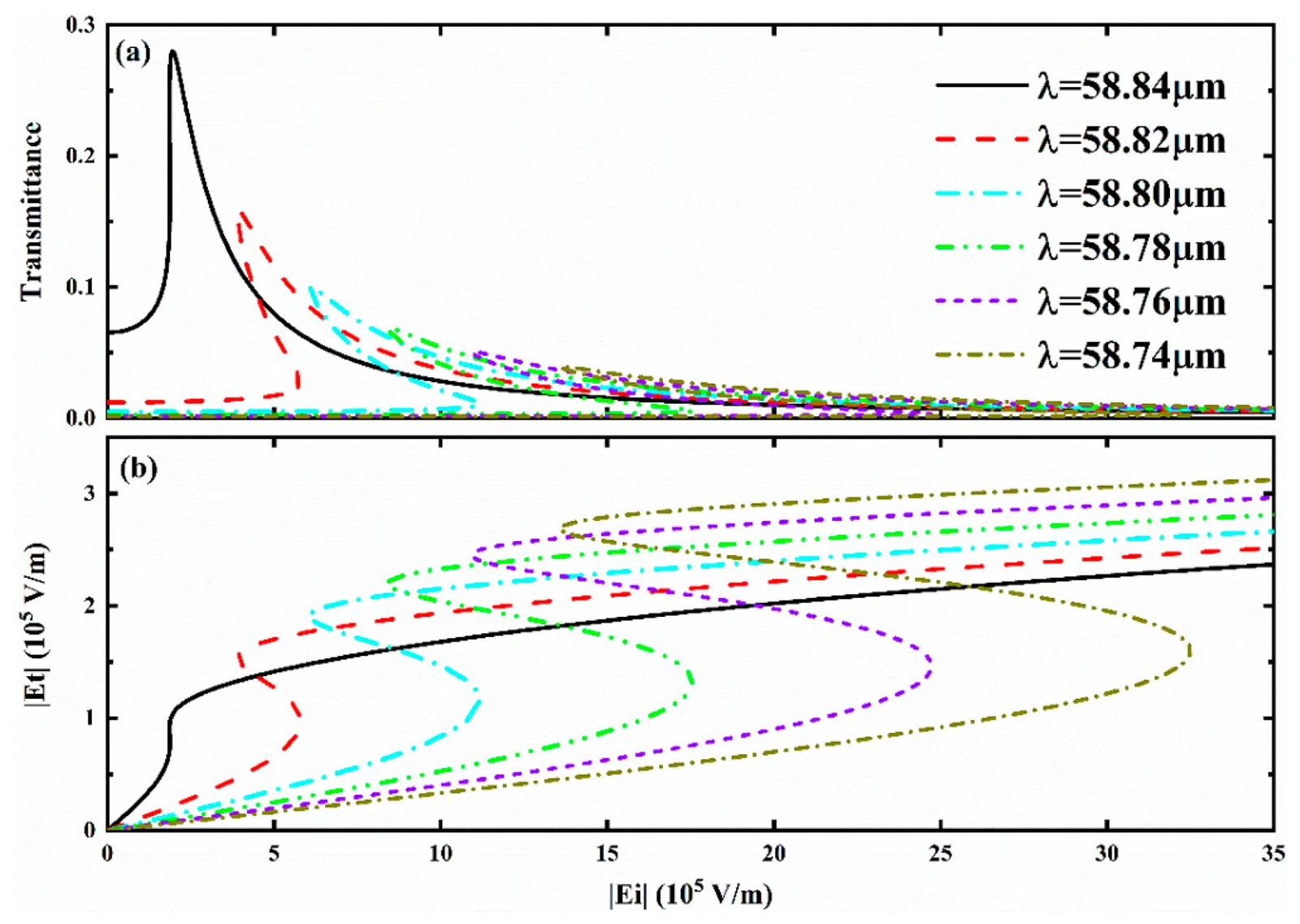
Dependence of (**a**) the transmittance and (**b**) transmitted electric field on the incident electric field for different incident wavelengths. Here, the incident angle is 0°, and the thickness of the WSM is 200 nm.

We previously discussed the influence of variations in the incident angle on the OB and demonstrated that the upper and lower thresholds, as well as the threshold width of the OB, can be tuned by adjusting the incident angle. Furthermore, we found that the OB is also influenced by the wavelength of the incident light. The regulatory effect of the incident wavelength on the OB is illustrated in Figure 5a,b.

When the incident wavelength λ exceeds 58.84 µm, no hysteresis loop is observed, indicating the absence of the OB phenomenon. As the wavelength decreases from 58.84 µm to 58.74 µm, a pronounced OB hysteresis appears, and the $|E_{i}{|}_{up}$ increases from $1.872\times 10^{5}$ V/m to $3.25\times 10^{6}$ V/m. The $|E_{i}{|}_{down}$ rises from $1.858\times 10^{5}$ V/m to $1.36\times 10^{6}$ V/m, and the $\Delta |E_{i}|$ increases from $1.4\times 10^{3}$ V/m to $1.89\times 10^{6}$ V/m, expanding by approximately 1350 times. Furthermore, as the incident wavelength λ decreases further, the OB threshold width exhibits a continuous increase. However, once the wavelength reaches a certain critical value, any further decrease causes the threshold width to decline gradually, eventually leading to the complete disappearance of the hysteresis loop.

**Figure 6:**
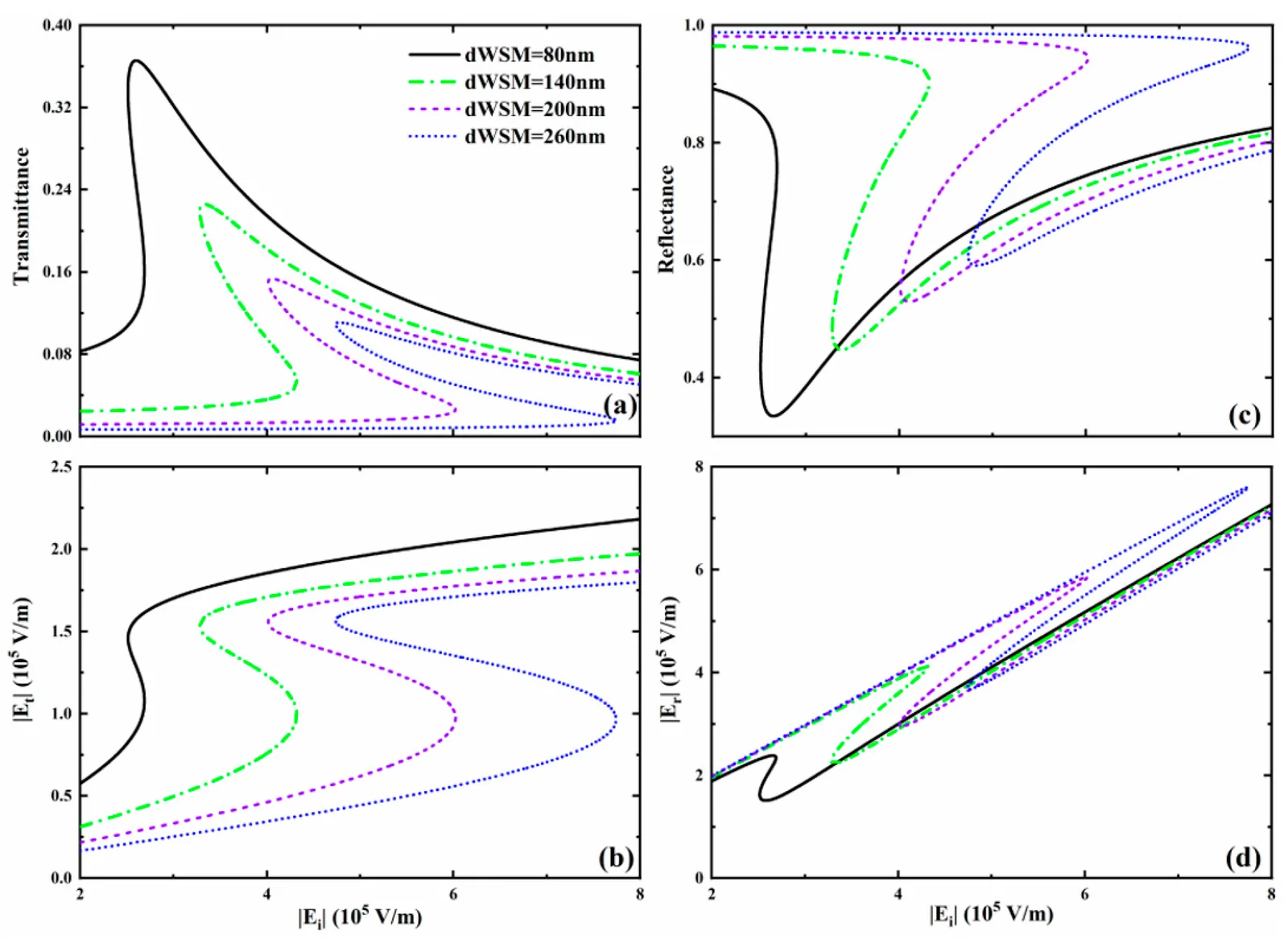
Influence of (**a**) transmittance, (**b**) transmitted electric field, (**c**) reflectance, and (**d**) reflected electric field on the incident electric field for different WSM thicknesses. The incident light has a frequency of 5.1004 THz and an incident angle of 0°.

To further investigate the dependence of OB characteristics on structural parameters in the composite structure, we theoretically studied the effect of WSM thickness on OB behavior. We focus on the influence of the thickness of WSM on OB, mainly considering the following aspects: firstly, as a nonlinear material, WSM plays a key role in the generation of OB, so we must have a very clear understanding of the influence and laws of WSM’s own conductivity and structural parameter changes on the hysteresis curve. Secondly, the thickness of WSM is the most important and intuitive parameter among WSM structural parameters. Although it is not possible to control OB by dynamically manipulating thickness changes, theoretically understanding the impact of thickness changes on hysteresis curves has a positive predictive effect on the trend effects that may occur due to small thickness changes caused by WSM preparation in reality. This is the main reason why we focus on the influence of thickness. Figure 6 illustrates the variation in transmittance (Figure 6a), transmitted electric field (Figure 6b), reflectance (Figure 6c), and reflected electric field (Figure 6d) with the incident electric field for different WSM thicknesses, under a fixed incident light frequency and incident angle. In Figure 6, it is evident that as the WSM thickness increases from 80 nm to 260 nm, the OB upper threshold $|E_{i}{|}_{up}$, lower threshold $|E_{i}{|}_{down}$, and threshold width $\Delta |E_{i}|$ all increase significantly. The increase in the OB threshold with WSM thickness can be physically understood as follows. A thicker WSM layer introduces stronger absorption and reduces the effective field enhancement at the interface. As a result, a higher incident field is required to achieve the same level of nonlinear refractive index modulation, leading to an increased OB threshold. This behavior indicates that the size of the OB hysteresis loop and the switching threshold can be effectively tuned by adjusting the WSM thickness. It is worth noting that although the absolute OB threshold increases with WSM thickness, the corresponding incident electric field amplitude remains on the order of 10^5^ V/m, as shown in Figure 6. This indicates that minor variations in WSM thickness do not lead to an order-of-magnitude change in the OB threshold or threshold width. The relative insensitivity of the threshold to thickness variations indicates a certain degree of robustness of the OB behavior against moderate structural variations.

**Figure 7:**
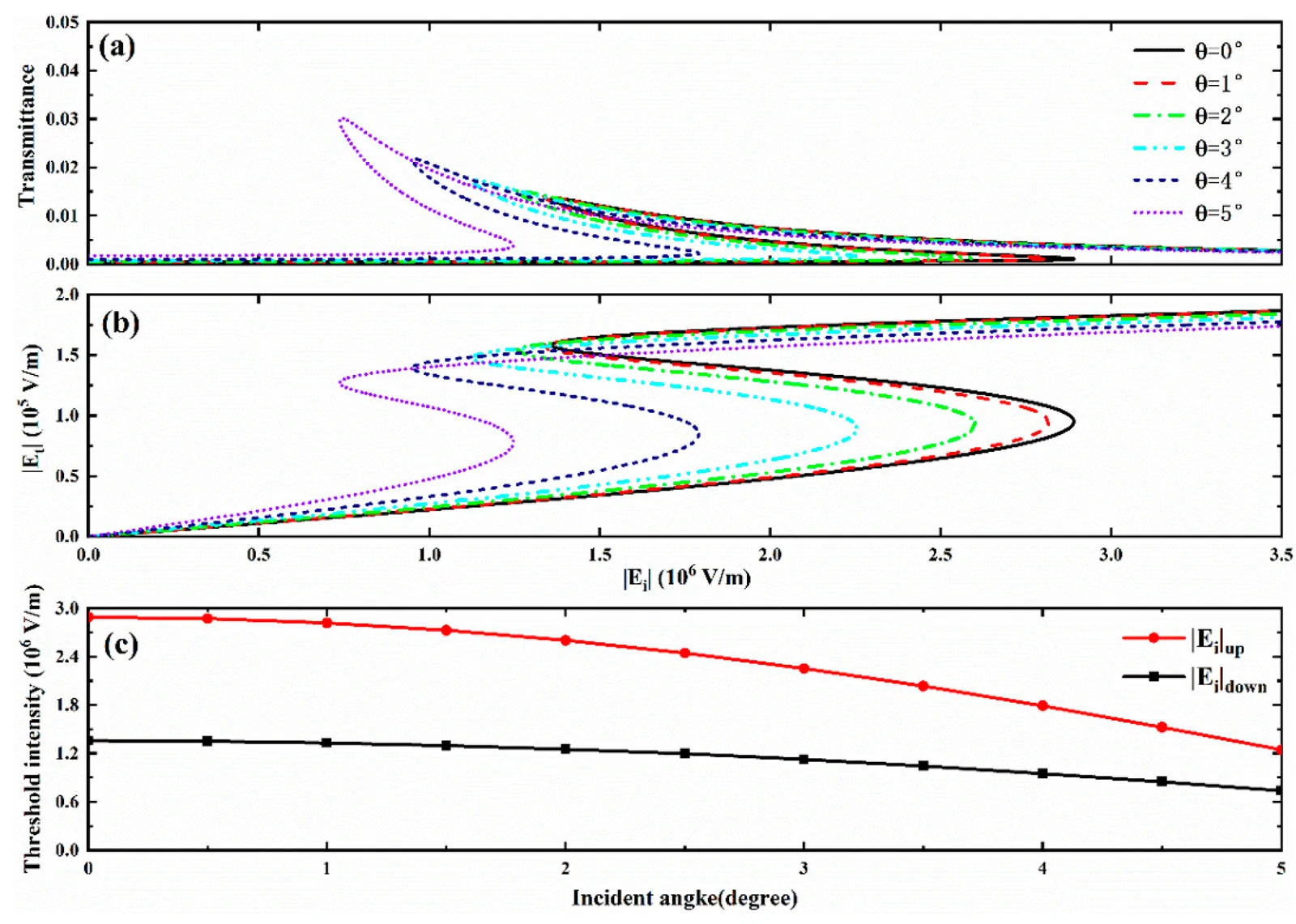
Dependence of (**a**) transmittance and (**b**) transmitted electric field on the incident electric field for different incident angles, and (**c**) influence of the incident angle on the upper (red curve) and lower (black curve) OB thresholds. The incident light frequency is 5.1004 THz, and the WSM thickness is 1 µm.

In previous work, we have shown that increasing the WSM thickness raises the OB upper threshold $|E_{i}{|}_{up}$, lower threshold $|E_{i}{|}_{down}$, and threshold width $\Delta |E_{i}|$ (Figure 6). This observation motivates further investigation into whether increasing the WSM thickness can also expand the range of incident angles over which OB remains stable. The motivation for this investigation arises from observations at a fixed WSM thickness of 200 nm (Figure 4): when the incident angle ranges from 0° to 2°, the system exhibits stable OB behavior; however, once the incident angle exceeds 3°, the OB phenomenon disappears entirely. This indicates that for relatively thin WSM layers, the OB is highly sensitive to variations in the incident angle, and its operational angular range is correspondingly limited. To test this hypothesis, we increased the WSM thickness from 200 nm to 1 µm and examined a wider range of incident angles. Figure 7a,b show the dependence of transmittance and transmitted electric field on the incident electric field for different incident angles after increasing the WSM thickness. As the incident angle increases from 0° to 5°, a clear and stable OB hysteresis curve is consistently observed. This behavior contrasts sharply with the case in Figure 4, where the OB remains stable only within an incident angle range of 0° to 2° for a WSM thickness of 200 nm. These results confirm our hypothesis that increasing the WSM thickness can effectively expand the range of incident angles over which OB occurs in the composite structure.

To quantitatively analyze the variation in the OB threshold with incident angle, we plotted the relationship in Figure 7c, with the incident angle on the horizontal axis and the OB threshold on the vertical axis. In this graph, the red and black curves represent the upper and lower OB thresholds, respectively. Both curves exhibit a monotonically decreasing trend as the incident angle increases. Furthermore, the difference between the two curves corresponds to the OB threshold width. As shown in Figure 7c, with increasing incident angle, the upper and lower threshold curves gradually converge, leading to a progressive reduction in the threshold width until it eventually vanishes.

Another important observation associated with the broadening of the incident angle range is that the incident electric field corresponding to the OB threshold changes by approximately one order of magnitude. When the WSM thickness is 200 nm (Figure 4b), the incident electric field at the OB threshold remains on the order of 10^5^ V/m. In contrast, in Figure 7, although the controllable incident angle range is expanded, the overall threshold incident electric field increases to the order of 10^6^ V/m. This behavior indicates that expanding the operational angular range by increasing the WSM thickness is achieved at the expense of a higher required switching power. Such a trade-off is an important factor that must be considered in the design of theoretical models. We compared the current scheme with other typical optical bistable schemes, as shown in Table 1. It is not difficult to see that our solution is competitive in terms of overall performance.

## 4. Conclusions

In this paper, we theoretically study the low-threshold and controllable OB in 1D PhC composite structures based on TESs and the synergistic nonlinear characteristics of WSM in the terahertz band. The results show that the local field enhancement effect of TESs and the excellent nonlinear characteristics of WSM play an important role in the generation of low-threshold OB. The upper and lower OB thresholds, as well as the threshold width, can be flexibly tuned by varying parameters such as the incident angle, incident wavelength, and WSM thickness. In particular, the OB threshold width can be tuned over a wide range by varying the incident wavelength. Furthermore, we find that modifying the WSM thickness not only enables effective regulation of the OB behavior but also alters the dependence of OB on the incident angle. We believe that this low-threshold, controllable OB scheme based on TESs, WSM, and 1D PhCs composite structures may provide theoretical guidance for future studies of nonlinear photonic structures.

## Figures and Tables

**Table 1:** Comparison between different optical bistable schemes.

Ref.	Mechanism	Structure	Threshold	Process Requirements	Frequency Range
[3]	Fano resonance	1D photonic crystal Fano resonance heterostructures	4.7 × 10^7^ V/m	High	Near Infrared
[12]	Optical Tamm State	Graphene- Bragg reflector structure	4.3 × 10^6^ V/m	High	THz
[21]	Defect mode	Asymmetrical 1D-PhCs	49.39 MW/m^2^	Moderate	THz
[27]	Conjugate Topological Edge State	Conjugate Topological Photonic Crystal	2 µW/m	Moderate	Near Infrared
[28]	Surface Plasmon Polariton	Otto Configuration	3.0 × 10^6^ V/m	High	THz
[29]	Cavity resonance	Photonic Crystals Cavity	1.0 × 10^6^ V/m	Moderate	THz
This work	Topological edge state	Photonic crystal heterostructure	3 × 10^5^ V/m	Moderate	THz

## Data Availability

The data that support the findings of this study are available from the corresponding author upon reasonable request.
